# Prevalence and Geographical Variation of Prothrombin G20210A Mutation in Patients with Cerebral Vein Thrombosis: A Systematic Review and Meta-Analysis

**DOI:** 10.1371/journal.pone.0151607

**Published:** 2016-03-31

**Authors:** Joaquín V. Gonzalez, Andrés G. Barboza, Fernando J. Vazquez, Esteban Gándara

**Affiliations:** 1 Hospital Universitario, Universidad Nacional de Cuyo, Mendoza, Argentina; 2 Division of neurology and neuro-intensive care, Hospital Central de Mendoza, Mendoza, Argentina; 3 Facultad de Ciencias Médicas, Universidad del Aconcagua, Mendoza, Argentina; 4 Internal Medicine Department, Hospital Italiano de Buenos Aires, Buenos Aires, Argentina; 5 Internal Medicine Research Unit, Internal Medicine Department, Hospital Italiano de Buenos Aires, Buenos Aires, Argentina; 6 Thrombosis Program, Division of Hematology-Department of Medicine, University of Ottawa-Ottawa Hospital, Ottawa, Canada; 7 Ottawa Hospital Research Institute, Ottawa, Canada; 8 School of Epidemiology, Public Health and Preventive Medicine, University of Ottawa-Ottawa Hospital, Ottawa, Canada; IIBB-CSIC-IDIBAPS, SPAIN

## Abstract

**Objectives:**

To compare the prevalence of prothrombin G20210A in patients with objectively confirmed cerebral vein or cortical vein thrombosis against healthy controls, and evaluate geographical variations.

**Design:**

Systematic review and meta-analysis of case control studies.

**Methods:**

We conducted a systematic review of electronic databases including MEDLINE and EMBASE. The main outcome was the prevalence of prothrombin G20210A in patients with objectively confirmed cerebral vein or cortical vein thrombosis; we also analyzed individual country variations in the prevalence. The random-effects model OR was used as the primary outcome measure.

**Results:**

In total 19 studies evaluated 868 cases of cerebral venous thrombosis and 3981 controls. Prothrombin G20210A was found in 103/868 of the patients with cerebral venous thrombosis and 105/3999 of the healthy controls [random effects pooled OR 5.838, 95% CI 3.96 to 8.58; I^2^17.9%]. The prevalence of prothrombin G20210A was significantly elevated in Italian studies (OR 9.69), in Brazilian studies (OR 7.02), and in German studies (OR 3.77), but not in Iranian studies (OR 0.98).

**Conclusion:**

Prothrombin G20210A is significantly associated with cerebral venous thrombosis when compared to healthy controls, although this association is highly dependent on the country of origin.

## Introduction

Cerebral venous thrombosis is rare thrombotic condition commonly associated with the presence of thrombophilia[1]. Prothrombin G20210A is one of the most common thrombophilias associated with venous thrombosis, including cerebral venous thrombosis[2]. In patients with deep vein thrombosis or pulmonary embolism the prevalence of prothrombin G20210A is highly dependent on the country of origin or ethnicity [3–7] but this variation has not been evaluated in patients with cerebral venous thrombosis. To address this issue, we examined the prevalence and geographical variation of carriers of the prothrombin G20210A in patients with cerebral vein or cortical vein thrombosis.

## Methods

We conducted a systematic review of electronic databases including MEDLINE and EMBASE to assess the prevalence of Prothrombin G20210A in patients with cerebral vein thrombosis (S1 Text). The timeframe of the search was from Jan 1995 to February 2015 and was designed with the support of a librarian from the Ottawa Hospital Health Services. The search was supplemented by hand-search of relevant articles, abstract books from international meetings and published reviews.

### Study Selection

Case control studies were included if they reported the prevalence of prothrombin G20210A in patients with objectively confirmed cerebral vein or cortical vein thrombosis. All potentially relevant articles were reviewed in full length to ensure that they satisfied the inclusion criteria: 1) enrolment of non-paediatric patients with cerebral vein or cortical vein thrombosis; 2) the study reported the diagnostic test used to confirm the diagnosis (including digital subtraction angiography, MRI, CT angiography or autopsy); 3) Prothrombin G20210A genotyping was available for all participants; 4) the numbers of cases and controls with and without prothrombin G20210A were provided in the article. Studies were excluded if their subjects did not receive objective testing for the prothrombin G20210A mutation, included a paediatric population or included patients in which the diagnostic methods was not reported or patient with self-reported cerebral venous thrombosis.

### Data Extraction and Quality Assessment

Two reviewers (J.G. and E.G.) independently assessed the eligibility of all articles identified in the initial search strategy and used the Newcastle–Ottawa Quality Assessment Scale for Observational Studies to assess the methodological quality of the selected studies. A third reviewer adjudicated all discrepancies if needed (A.B.)

### Outcome Measure

The primary outcome measure was the odds ratio (OR) for the prevalence of prothrombin G20210A patients with cerebral vein or cortical vein thrombosis as compared with healthy controls. We also aimed to analyze the prevalence of prothrombin G20210A mutation across different countries, if two or more studies were available.

### Data Synthesis and Analysis

The meta-analysis was conducted in accordance with the guidelines of the Preferred Reporting Items for Systematic Reviews and Meta-analyses (PRISMA) statement (S1 and S2 Tables presents the meta-analysis of genetic association studies checklist). The random-effects model OR was used as the outcome measure, along with the corresponding 95% confidence intervals (CIs). The I^2^ statistic was used to quantify heterogeneity among the pooled estimates across studies. An I^2^ value less than 25% was considered low-level heterogeneity, 25% to 50% as moderate-level, and greater than 50% as high-level. Homozygote and heterozygote carriers of prothrombin G20210A were analyzed as one group due to the rarity of homozygote carriers. A funnel plot and test for bias using the Harbord and Egger test[8]. We also added a L'Abbé plot which is useful for exploring heterogeneity and identifying outlying trials in a meta-analysis[9]. The statistical analysis and graphs were performed using MedCalc Statistical Software version 12.7.7 (MedCalc Software, Ostend, Belgium; http://www.medcalc.org; 2013) and the bias analysis was performed with STATS direct (StatsDirect Ltd. StatsDirect statistical software. http://www.statsdirect.com. England: StatsDirect Ltd. 2013).

## Results

**Fig 1:**
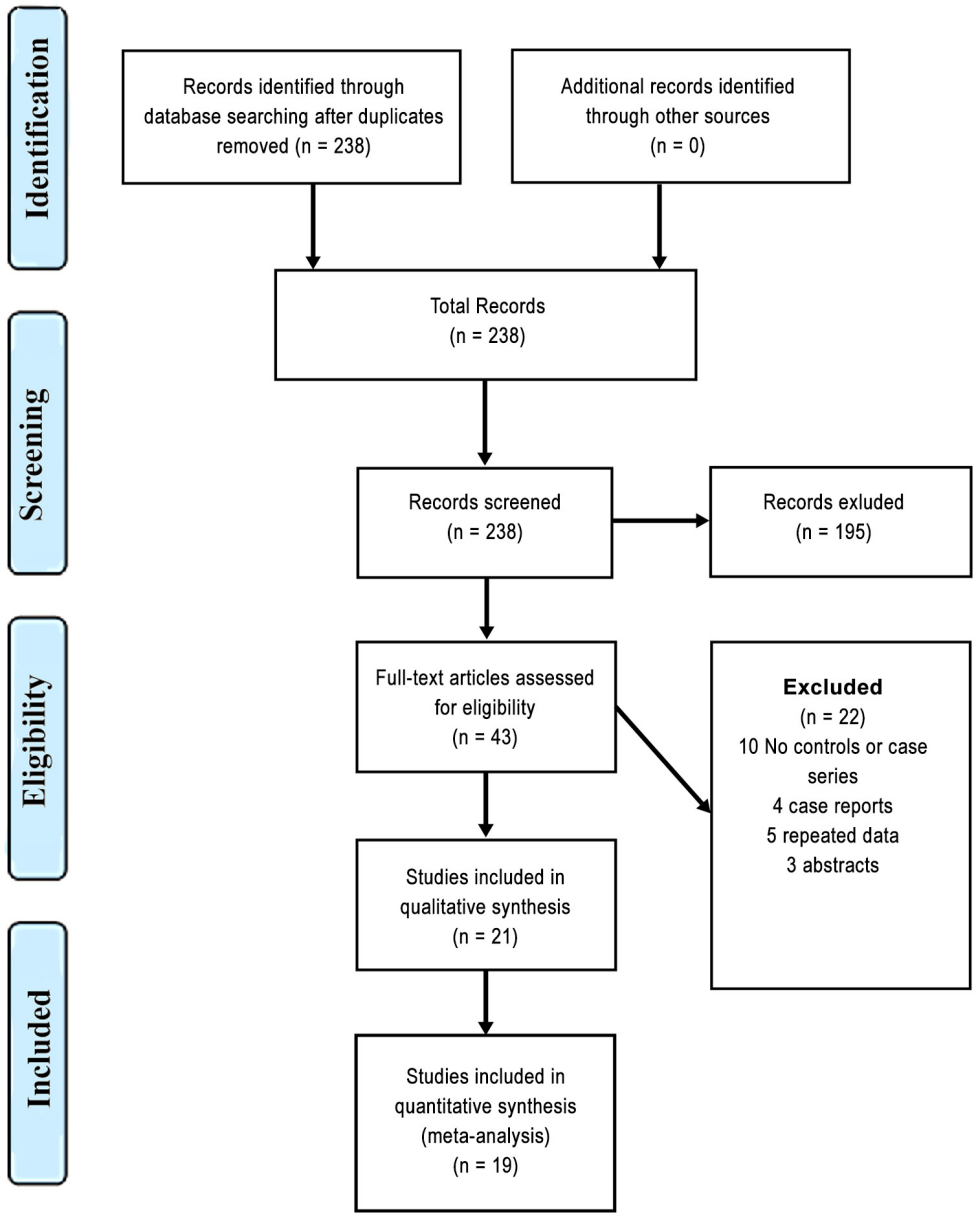
Flow diagram.

As shown in fig 1 our initial electronic search identified 238 relevant abstracts (after removal of duplicates). One hundred and ninety five were considered non-relevant to the search and excluded. Of the 43 that were selected for full text review 21[10–30]were included in this review, 19[10–12,14,16–25,27–31]of which were used for the primary analysis (in Table 1 we present the studies characteristics and in Table 2 their quality assessment); 22 were excluded for the reasons described in Fig 1. Two studies were excluded from the primary analysis as they did not report the method used for the diagnosis of cerebral venous thrombosis[13,26]. Five of the studies were conducted in Italy[17,21,27,29,31], three in Brazil[18,25,28], three in Germany[10,20,24], two in Iran[16,23], one in France[19], one in India[32],one in Netherlands[30]one in Switzerland[11], one in Tunisia[14] and one in the United Kingdom[12].

**Table 1:** Characteristics of the studies included.

Study	Year	Country	Diagnostic method	N CVT	N Controls	Matched?	Hardy-Weinberg equilibrium assessed?	% pregnancy CVT	% OCP CVT	% Female with CVT
**Ashjazadeh**[16]	2012	Iran	MRI and MRV	57	50	Yes, age/gender/ethnicity	No	7	47	66
**Ben Salem-Berrabah**[14]	2012	Tunisia	CT scan, MRI/MRV or autopsy	26	197	No	Yes, in equilibrium	.	.	80
**Bombeli**[11]	2002	Switzerland	CT scan, MRI/MRV or autopsy	51	120	No	No	.	3.2	76.5
**Boncoraglio**[17]	2004	Italy	Angiography, CT or MRI	26	100	No, healthy hospital workers	No	.	42	73
**Colaizo**[29]	2007	Italy	Digital angiography, CT or MRI	45	286	sex, age and social status	Yes	NA	NA	69%
**Gadelha**[18]	2005	Brazil	Angiography or MRI	21	217	Age/racial background, no history of thrombosis or genetic relationship	No	.	85%	84
**Hillier**[12]	1998	UK	Digital angiography, CT, autopsy or MRI	15	300	No	No	.	38	70
**Koopman**[30]	2009	Netherlands	Digital angiography, CT, surgery or MRI	19	19	Age and Sex	No	20%	60%	79%
**Le Cam-Duchez**[19]	2005	France	Angiography or MRI	26	84	Age and sex	No	9	44	69
**Lichy**[20]	2005	Germany	MRI and/or angiography	77	203	No	No	11.30%	43%	78%
**Madonna**[21]	2000	Italy	MRI and/or angiography	10	254	Sex and age	No	.	33	60%
**Martinelli** [31]	2003	Italy	MRI, CT and/or angiography	121	242	Sex, age, geographic origin, and level of education	No	NA	96%	74%
**Nagaraja**[32]	2007	India	MRI/MRV	96	103	Age	No	100	0	100
**Rahimi**[23]	2010	Iran	MRI	24	100	Age/ gender; Kurdish descent	No	.	.	70%
**Reuner**[10]	1998	Germany	MRI and/or angiography	45	354	No	No	10	85	75%
**Ringelstein**[24]	2012	Germany	MRI and/or angiography	136	1054	No, but same ethnicity	Not done for prothrombin gene	.	NR	34
**Rodrigues**[25]	2004	Brazil	MRI and/or angiography	42	134	No	No	20	60	67
**Ventura**[27]	2004	Italy	MRI, CT and/or angiography	30	40	Age/gender	No		9	53
**Voetsch**[28]	2000	Brazil	MRI, CT and/or angiography	14	225	Age/gender	No	.	.	71
**Excluded from main analysis**										
**Tufano**[26]	2014	Italy	Not reported	56	184	Age/gender same ethnicity		NR	53.7	73%
**Margaglione**[13]	2001	Italy	Not reported	28	1304	No		.	.	.

CVT: Cerebral venous thrombosis; OCP: Oral contraception; CT: computerized axial tomography; MRI/MRV: Magnetic resonance imaging (MRI) or magnetic resonance venography; Angiography: digital subtraction angiography

**Table 2:** Quality assessment.

Study	Is the case definition adequate?	Representativeness of the cases	Selection of Controls	Definition of Controls	Comparability of cases and controls on the basis of the design or analysis	Ascertainment of exposure	Same method of ascertainment for cases and controls	Non-Response rate
**Ashjazadeh**[16]	*	*	*	*		*	*	*
**Ben Salem-Berrabah**[14]	*	*				*	*	*
**Bombeli**[11]	*	*	*	*		*	*	*
**Boncoraglio**[17]	*	*				*	*	*
**Colaizo**[29]	*	*	*	*		*	*	*
**Gadelha**[18]	*	*	*	*		*	*	*
**Hillier**[12]	*	*	*	*		*	*	*
**Koopman**[30]	*	*				*	*	*
**Le Cam-Duchez**[19]	*	*	*	*		*	*	*
**Lichy**[20]	*	*	*	*		*	*	*
**Madonna**[21]	*	*	*	*		*	*	*
**Martinelli** [31]	*	*	*			*	*	*
**Nagaraja**[32]	*	*	*	*		*	*	*
**Rahimi**[23]	*	*				*	*	*
**Reuner**[10]	*	*	*	*		*	*	*
**Ringelstein**[24]	*	*	*	*		*	*	*
**Rodrigues**[25]	*	*	*	*		*	*	*
**Ventura**[27]	*		*			*	*	*
**Voetsch**[28]	*	*	*	*		*	*	*

**Fig 2:**
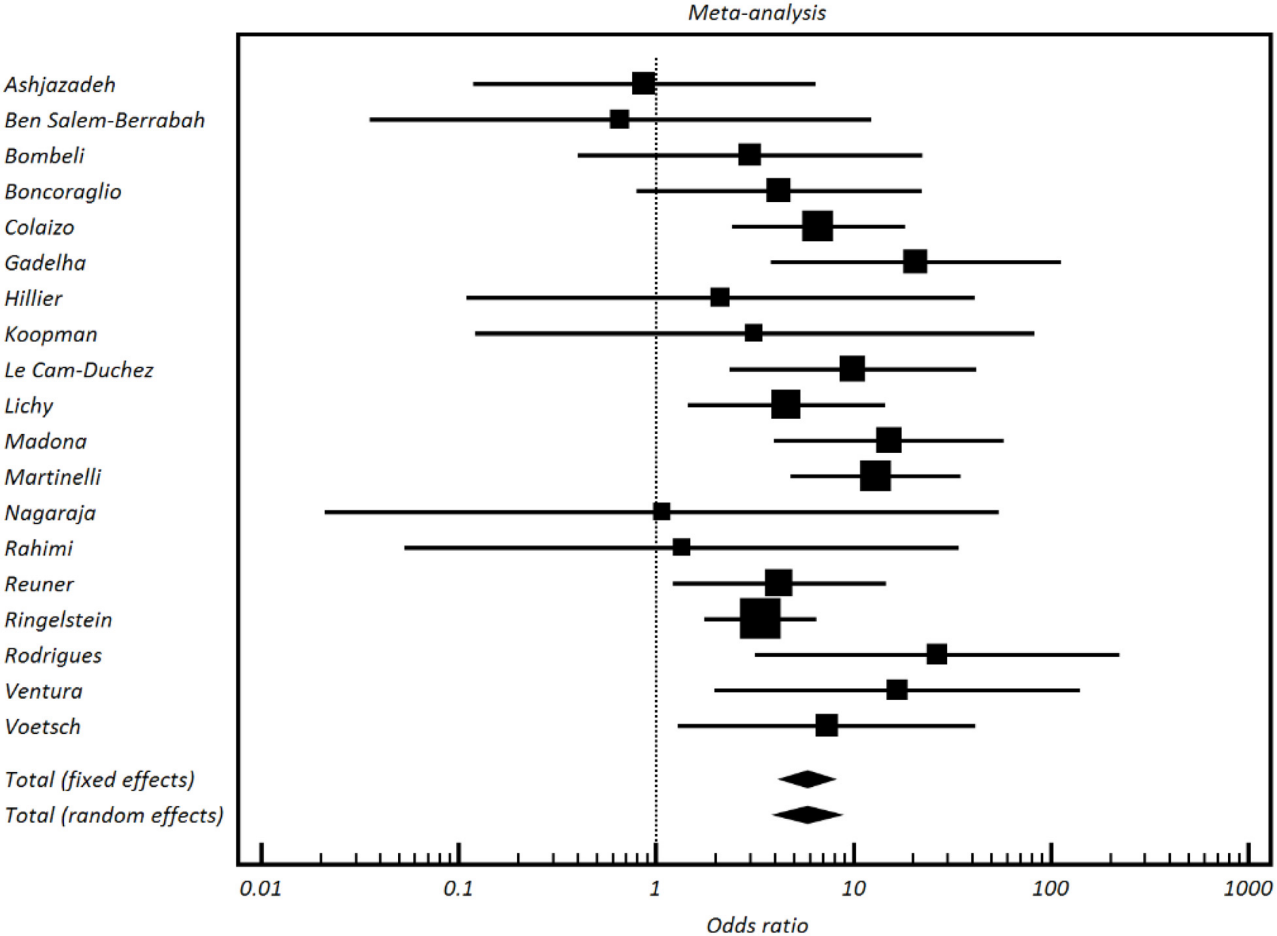
Forest plot prevalence of prothrombin G20210A.

**Fig 3:**
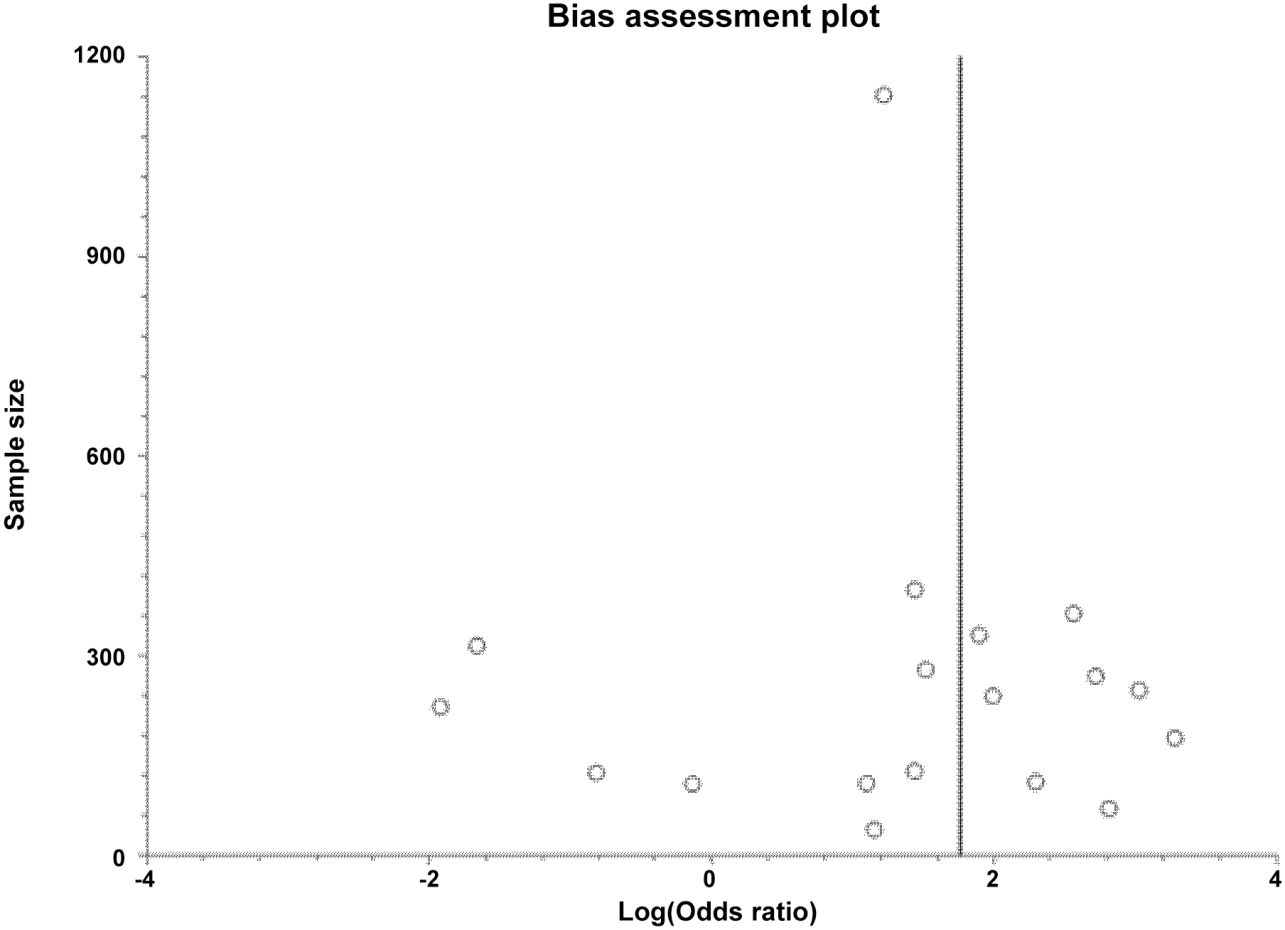
Bias assessment plot.

**Fig 4:**
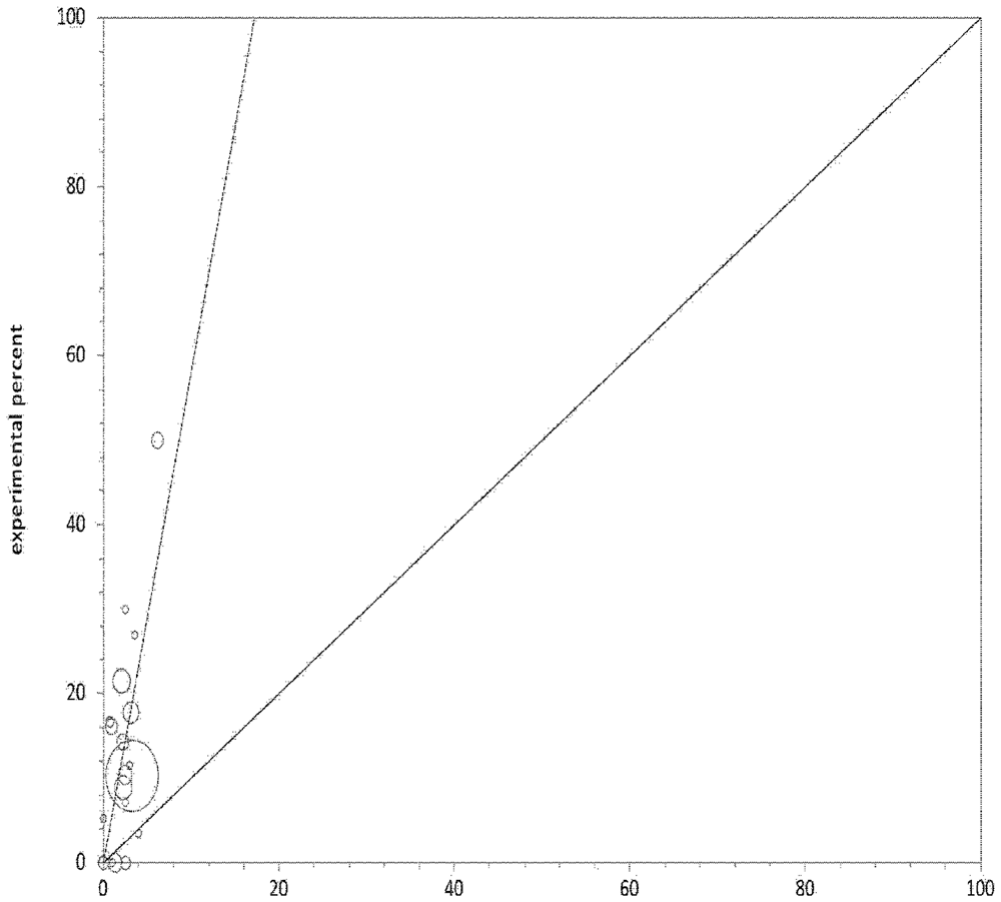
L’Abbé bias plot.

In total the 19 studies evaluated 868 cases of cerebral venous thrombosis and 3999 controls (see Table 3 for a more comprehensive review of the number of patients included in each study individual OR and bias assessment). Prothrombin G20210A was found in 103/868 of the patients with cerebral venous thrombosis and 105/3999 of the controls [random effects pooled OR 5.838, 95% CI 3.96 to 8.58; I^2^17.9%] (Fig 2) with low evidence of bias (Figs 3 and 4). As shown in Table 3 the addition of the two relevant studies not reporting the diagnostic methods used did not modify the random effects pooled OR[13,26].

**Table 3:** Meta-analysis and individual study data.

**Study**	**Carriers of PTGM**^/CVT* **cases**	**Carriers of PTGM**^**/Healthy controls**	**Odds ratio**	**95% CI**
Ashjazadeh	2/57	2/50	0.87	0.11 to 6.43
Ben Salem-Berrabah	0/26	5/197	0.66	0.035 to 12.28
Bombeli	2/28	2/80	3.00	0.40 to 22.38
Boncoraglio	3/26	3/100	4.21	0.79 to 22.26
Colaizo	8/45	9/286	6.65	2.41 to 18.31
Gadelha	5/31	2/217	20.67	3.81 to 111.99
Hillier	0/15	4/300	2.12	0.10 to 41.26
Koopman	1/19	0/19	3.16	0.12 to 82.64
Le Cam-Duchez	7/26	3/84	9.94	2.35 to 42.063
Lichy	8/77	5/202	4.56	1.44 to 14.43
Madona	5/10	16/259	15.18	3.98to 57.93
Martinelli	26/121	5/242	12.97	4.83 to 34.78
Nagaraja	0/96	0/103	-	
Rahimi	0/24	1/100	1.35	0.053 to 34.25
Reuner	4/45	8/354	4.22	1.21 to 14.62
Ringelstein	14/136	33/1007	3.38	1.76 to 6.50
Rodrigues	7/42	1/134	26.60	3.167 to 223.42
Ventura	9/30	1/40	16.71	1.98 to 141.07
Voetsch	2/14	5/225	7.33	1.28 to 41.77
**Total (random effects)**	**103/868**	**105/3999**	**5.83**	**3.96 to 8.58**
**I**^**2**^ **(inconsistency)**			17.9%	**0.00 to 53.41**
Bias indicators. Harbold-Egger: bias = -0.30 (92.5% CI = -2.05 to 1.46) P = 0.74				
**Analysis with excluded studies**	**Carriers of PTGM**^/CVT* **cases**	**Carriers of PTGM**^**/Healthy controls**	**Odds ratio**	**95% CI**
Margaglione	6/28	56/1301	6.06	2.36 to 15.54
Tufano	16/55	10/183	7.09	2.99 to 16.82
**Total (random effects)**	125/951	171/5483	5.93	4.32 to 8.13
**I**^**2**^ **(inconsistency)**			8.09%%	**0.00 to 43.53**
Bias indicators. Harbold-Egger: bias = -0.21 (92.5% CI = -1.83to 1.41) P = 0.80				

^PTGM: prothrombin G20210A;

*CVT: cerebral vein thrombosis

As shown in Table 4, in Italian studies [17,21,27,29,31] prothrombin G20210A was found in 51/232 of patients with cerebral venous thrombosis and in 34/927 of the controls (random effects pooled OR 9.69, 95% 5.51 to 17.05; I^2^ 0%). In Brazilian studies[18,25,28] prothrombin G20210A was found in 14/82 of patients with cerebral venous thrombosis and in 14/576 of the controls (random effects pooled OR 7.02, 95% CI 2.79 to 17.63; I^2^ 21.8%). In the three German[10,20,24] studies prothrombin G20210A was found in 28/255 of patients with cerebral venous thrombosis and in 49/1563 of the controls (random effects pooled OR 3.77, 95% CI 2.22 to 6.26; I^2^ 0%). In the two Iranian studies[16,23] there was no difference in the prevalence prothrombin G20210A between patients with cerebral venous thrombosis and controls (random effects pooled OR 0.98, 95% CI 0.18 to 5.39; I^2^ 0%).

**Table 4:** Individual country meta-analysis.

**Study**	**Carriers of PTGM**^/CVT* **cases**	**Carriers of PTGM**^**/Healthy controls**		**Odds ratio**	**95% CI**
**Italy**					
Boncoraglio	3/26	3/100		4.21	0.79 to 22.26
Colaizzo	8/45	9/286		6.65	2.41 to 18.31
Madona	5/10	16/259		15.18	3.98 to 57.93
Martinelli	26/121	5/242		12.97	4.83 to 34.78
Ventura	9/30	1/40		16.71	1.98 to 141.07
**Total (random effects)**	**51/232**	**34/927**		**9.69**	**5.51 to 17.05**
**I**^**2**^ **(inconsistency)**				**0.00%**	**0.00 to 64.1**
Bias indicator. Horbold-Egger: bias = 1.8 (92.5% CI = -2.96to 6.63) P = 0.38					
**Brazil**					
Gadelha	5/26	5/217		10.0	2.70 to 37.72
Rodrigues	7/42	7/134		3.62	1.19 to 11.03
Voetsch	2/14	2/225		18.58	2.40 to 143.52
**Total (random effects)**	**14/82**	**14/576**		**7.02**	**2.79 to 17.63**
**I**^**2**^ **(inconsistency)**				**22.82%**	**0.00 to 97.41**
Bias indicator. Horbold-Egger: bias = 1.37 (92.5% CI = -20.93 to 23.68) P = 0.69					
**Germany**	**Carriers of PTGM**^/CVT* **cases**	**Carriers of PTGM**^**/Healthy controls**			
Lichy	10/77	5/202		5.88	1.94 to 17.82
Reuner	4/45	8/354		4.22	1.21 to 14.62
Ringelstein	14/136	33/1007		3.38	1.76 to 6.50
**Total (random effects)**	**28/255**	**49/1563**		**3.77**	**2.22 to 6.26**
**I**^**2**^ **(inconsistency)**				**0.00%**	**0.00 to 72.29**
Bias indicators. Horbold-Egger: bias = 0.93 (92.5% CI = -3.34 to 5.21) P = 0.31					
**Iran**	**Carriers of PTGM**^/CVT* **cases**	**Carriers of PTGM**^**/Healthy controls**			
Ashjazadeh	2/57	2/50		0.87	0.11 to 6.43
Rahimi	0/24	1/100		1.35	0.05 to 34.25
**Total (random effects)**	**2/81**	**3/150**		**0.98**	**0.18 to 5.39**
**I**^**2**^ **(inconsistency)**				**0.00%**	**0.00 to 0.00**
Bias indicators not done due to low number of studies					

^PTGM: prothrombin G20210A;

*CVT: cerebral vein thrombosis

## Discussion

Our systematic review confirmed a significant association of prothrombin G20210A with cerebral venous thrombosis but this association is highly dependent to the country of origin. Individual country meta-analysis suggested significant association between prothrombin G20210A and cerebral venous thrombosis in studies conducted in Italy[17,21,27,31], Brazil[18,25,28] and Germany[10,20,24]. Consistently with prior literature[3] suggesting a low prevalence of the condition in those from Asian or African descent, none of the studies included from India[33], Iran[16,23] or Tunisia[14]reported an association between prothrombin G20210A and cerebral venous thrombosis. For the first time, and to the best of our knowledge, information about the geographical variations of prothrombin G20210A with cerebral venous thrombosis has been presented.

Similar to deep vein thrombosis and pulmonary embolism the question that remains unanswered is the value of screening for prothrombin G20210A with cerebral venous thrombosis in order to predict who is at high risk of recurrent venous thromboembolism? Our results would suggest that given it high prevalence in countries such as Italy or Brazil, it would be reasonable to screen for this condition, especially in those with unprovoked events[34]. On the other hand, evidence suggests that the rate of recurrent venous thromboembolism associated with prothrombin G20210A gene mutation is low in those with deep vein thrombosis or pulmonary embolism[35,36]. Two our knowledge only two studies, conducted in Italy, have reported the rate of recurrent venous thromboembolism in patients with prothrombin G20210A and cerebral venous thrombosis who discontinue anticoagulation[26,37], none of them showed an increased risk for recurrent venous thromboembolism in carriers of prothrombin G20210A diagnosed with cerebral venous thrombosis.

Other systematic reviews have addressed the prevalence of prothrombin G20210A in patients diagnosed with cerebral venous thrombosis[2,38]. Our results confirms prior findings in other systematic reviews[2,38] suggesting a significant association between prothrombin G20210A diagnosed with cerebral venous thrombosis and provide. Also for the first time information about geographical variations associated with the prevalence of prothrombin G20210A in patients with cerebral vein thrombosis is presented. As previously suggested in patients with deep vein thrombosis or pulmonary embolism[3–7], we found that the prevalence of prothrombin gene mutation was higher in southern European countries, and no association in Asian countries.

Our systematic review has limitations. First, we did could not evaluate the prevalence of prothrombin gene mutation in patients with unprovoked cerebral venous thrombosis. Second, not all the studies used matching and, as such, potentially introduced bias. Third, homozygous prothrombin G20210A could not be assessed owing to the small number of patients affected. Fourth, our individual country analysis did not take into different genetic populations who might live in a country[39]. Fifth, we did not adjust for important risk factors (such as provoked cerebral vein thrombosis vs. unprovoked; female vs. male) which could introduce some bias. Finally, most of the studies included did not reported on the Hardy-Weinberg equilibrium and given that only two of the studies reported on it we did not conducted a sensitivity analysis[40,41].

In conclusion, our systematic review confirmed a significant association of prothrombin G20210A with cerebral venous thrombosis but suggest that this association is highly dependent to the country of origin. More studies should evaluate the role of prothrombin G20210A as a predictor of recurrent venous thromboembolism in patients with cerebral venous thrombosis, especially in those countries where its prevalence high prevalence.

## Supporting Information

S1 Table 2009 PRISMA checklist. (DOC)

S2 Table Meta-analysis of genetic association studies checklist. (DOCX)

S1 Text Literature search. (PDF)
