# Supplementary material for: Prevalence and Geographical Variation of Prothrombin G20210A Mutation in Patients with Cerebral Vein Thrombosis: A Systematic Review and Meta-Analysis
Source: PLoS One. 2016 Mar 31;11(3):e0151607. doi: 10.1371/journal.pone.0151607 (PMC4816324; doi:10.1371/journal.pone.0151607)
Supplement: S1 Text — (PDF) [file pone.0151607.s003.pdf]

Database: Embase Classic+Embase <1947 to 2015 March 02>, Ovid MEDLINE(R) In-Process & Other Non-Indexed Citations and Ovid MEDLINE(R) <1946 to Present>
Search Strategy: **March 3, 2015**

----

1 Prothrombin/ (30405)
2 prothrombin.tw. (53922)
3 (G20210A or "20210" or 20210A).tw. (4705)
4 1 or 2 or 3 (64672)
5 ((sinus and thrombosis) or (thrombosis and cerebral and (venous or vein or sinus))).ti. or exp Sinus Thrombosis, Intracranial/ or (intracranial and thrombosis).ti. (12221)
6 4 and 5 (348)
7 limit 6 to english language (300)
8 remove duplicates from 7 (226)
9 8 use prmz (90)
10 8 use emczd (136)
11 from 8 keep 1-226 (226)

Europe Pubmed Central : **March 3, 2015**

(ABSTRACT:"cerebral" AND ABSTRACT:"thrombosis" AND ABSTRACT:"venous") OR (ABSTRACT:"cerebral" AND ABSTRACT:"thrombosis" AND ABSTRACT:"vein") OR (ABSTRACT:"intracranial" AND ABSTRACT:"thrombosis") OR (ABSTRACT:"sinus" AND ABSTRACT:"thrombosis") AND (ABSTRACT:"G20210A" OR ABSTRACT:"20210A" OR ABSTRACT:"20210" OR ABSTRACT:"Prothrombin") AND LANG:eng
